# In Vitro Ocular Cytotoxicity of BEE Venom in ARPE-19, NTM5, HCET, and HCEC-12 Cell Lines

**DOI:** 10.1007/s12013-026-02042-y

**Published:** 2026-03-11

**Authors:** Emine Kubra Bilir, Hidayet Tutun, Sedat Sevin, Şeyda Kıvrak, Tommaso Bucci, Rachel Williams

**Affiliations:** 1 Department of Eye and Vision Science, Institute of Life Courses and Medical Sciences, University of Liverpool, Liverpool, UK; 2 Department of Pharmacology and Toxicology, Faculty of Veterinary Medicine, Burdur Mehmet Akif Ersoy University, Burdur, Türkiye Turkey; 3 Department of Pharmacology and Toxicology, Faculty of Veterinary Medicine, Ankara University, Ankara, Türkiye Turkey; 4 Department of Nutrition and Dietetics, Faculty of Health Sciences, Muğla Sıtkı Koçman University, Muğla, Türkiye Turkey; 5 Liverpool Centre for Cardiovascular Science, Institute of Life Courses and Medical Sciences, University of Liverpool, Liverpool, UK

**Keywords:** Bee venom, Human ocular cell lines, Ocular toxicity, HPLC, Apis mellifera

## Abstract

Bee venom (BV) is a complex natural secretion of *Apis mellifera* with documented anti-inflammatory, antimicrobial, and anticancer properties. Despite its therapeutic potential, safety concerns remain due to its strong cytotoxic effects, particularly in sensitive tissues such as the eye. In this study, we evaluated the composition and ocular cytotoxicity of purified BV obtained from the Muğla ecotype of *Apis mellifera anatoliaca*. High-performance liquid chromatography with variable wavelength detection (HPLC-VWD) confirmed the presence of three principal components; melittin (71.08 ± 0.33%), phospholipase A₂ (12.98 ± 0.27%), and apamin (3.02 ± 0.25%) at retention times consistent with reference standards. Cytotoxicity was assessed in four human ocular cell lines: ARPE-19 (retinal pigment epithelium), NTM5 (trabecular meshwork), HCE-T (corneal epithelium), and HCEC-12 (corneal endothelium), using MTT viability assays and live/dead staining over 24 h and 48 h. BV exerted strong, dose and time dependent cytotoxic effects across all cell lines, with ARPE-19 and NTM5 cells exhibiting the highest sensitivity. IC₅₀ values were 22.36 µg/mL (24 h) and 12.55 µg/mL (48 h) in ARPE-19, and 20.78 µg/mL (24 h) and 13.07 µg/mL (48 h) in NTM5 cells. By contrast, HCEC-12 and HCE-T cells displayed moderate sensitivity, with IC₅₀ values between 29 and 34 µg/mL. These findings highlight tissue-specific differences in BV susceptibility. While BV may offer therapeutic promise, its narrow safety margin in ocular cells underscores the importance of controlled delivery strategies to minimize toxicity. This study provides foundational data for evaluating the ocular safety profile of BV and informs future translational applications.

## Introduction

Bee venom (BV), or apitoxin, is a natural secretion of worker bees (*Apis mellifera* L.) that has been valued in traditional medicine for centuries and is increasingly studied in modern biomedical research [1]. It contains a diverse mixture of bioactive peptides, enzymes, and small molecules, with melittin, phospholipase A2 (PLA2), apamin, and hyaluronidase recognised as its most abundant and biologically active components [2]. These compounds are responsible for a wide spectrum of pharmacological activities, including anti-inflammatory [3], neuroprotective [4, 5], anticancer [6], cytotoxic [4], antibacterial [7], wound healing [8] attributed to these compounds.

The Anatolian honeybee (*Apis mellifera anatoliaca*), particularly the Muğla/Türkiye ecotype, represents a geographically unique subspecies with distinct venom composition [9]. Studies have shown that venom from Anatolian honeybee venom exhibits notable anti-inflammatory activity by suppressing pro-inflammatory cytokine production in mammalian macrophages, while maintaining limited cytotoxicity at therapeutic concentrations [3]. Moreover, cytotoxic effects of *Apis mellifera anatoliaca* venom against human lung adenocarcinoma (Calu-3) cells have demonstrated dose-dependent growth inhibition and suppression of cellular migration, highlighting its potential as an anticancer agent while emphasizing the importance of precise dosing [9].

Accurate characterization of BV is essential for understanding its biological properties. High-performance liquid chromatography (HPLC) remains the gold standard for analysing venom peptides and proteins, providing detailed profiles of melittin, PLA2, and other pharmacologically relevant compounds [10, 11]. Such analytical approaches are critical when assessing purified venom fractions for cytotoxicity in mammalian cell systems [12]. Previous studies have reported that purified BV and its components can induce apoptosis, necrosis, or combined cytotoxic responses depending on dose and cell type, with synergistic interactions between melittin and PLA2 further amplifying cytotoxic potential [13].

Despite promising therapeutic applications, systemic or topical use of BV can cause a variety of adverse effects, ranging from mild local skin reactions to life-threatening anaphylaxis. Adverse outcomes are influenced by dosage, route of administration, and tissue sensitivity [14]. Clinical reports describe ocular complications after bee stings, including conjunctivitis, cataract, uveitis, optic neuropathy, corneal edema, and endothelial toxicity [15, 16]. Experimental evidence further suggests that intravitreal injection or topical exposure to BV can produce ocular toxicity, highlighting the need for rigorous cytotoxicity and dose response studies [14, 17].

The eye is one of the most sensitive organs to toxicity [18]. The cornea and retina are essential for visual function, and their damage can lead to severe or irreversible outcomes, including Fuchs’ corneal dystrophy and retinopathy [19, 20]. Drug delivery to such sensitive tissues requires careful evaluation, and in vitro models provide a critical platform for early-stage testing before preclinical development ([21, 22]; Dunn et al., 1996). Cell lines such as ARPE-19 (retinal pigment epithelium), NTM5 (trabecular meshwork) [23], HCE-T (human corneal epithelium) [24], and HCEC-12 (corneal endothelium) [25–27] are widely used models to investigate tissue-specific responses to potential therapeutics and toxins.

Determination of the half-maximal inhibitory concentration (IC50) of BV in various ocular cells provides valuable insight into inter-tissue sensitivity, toxicity thresholds, and safe therapeutic ranges. Such information is critical for guiding controlled-release and targeted delivery strategies to minimize toxicity. However, studies specifically evaluating the effects of BV on ocular cell models remain scarce. Therefore, this study aimed to purify crude bee venom collected from the Muğla ecotype of *Apis mellifera anatoliaca* using HPLC and to comparatively assess its cytotoxic effects on ARPE-19, NTM5, HCE-T, and HCEC-12 cells. By addressing this gap, we sought to establish foundational data on the ocular safety profile and therapeutic potential of Anatolian bee venom.

## Materials and Methods

### Sample Collection and Preparation of bee Venom

Bee venom samples were collected from *Apis mellifera anatoliaca* (Muğla ecotype) hives in the Afyon-Sandıklı districts of Türkiye during the *Cistus salviifolius* (Laden) pollen harvest season between June and July 2024. A bee venom electroshock collector (BeeSas beekeeping, Turkey) was used to collect the samples.

After the 20-minute shock treatment, the bee venom residue solidified on the glass plate surface, and it was left in a shaded, ventilated area for 8 h. The venom was then scraped off with a sharp scalpel to obtain the venom. The resulting bee venom was freeze-dried using a freeze-dryer and stored in a -18 °C freezer until analysis.

### Preparation of bee Venom for the Analyses

In order to obtain purified bee venom samples, 3 g of collected crude bee venom was dissolved in 1 L of 20% ethanol solution. Ultrasonic extraction was performed in sweep mode for 30 min, followed by centrifugation at 5000 rpm and 4 °C for 10 min. The supernatant was filtered through a 0.22 μm pore size polytetrafluoroethylene (PTFE) membrane to remove particulate matter, then lyophilised at − 86 °C and stored in tightly sealed containers protected from light at − 18 °C until analysis.

### Determination of Content and Composition of bee Venom

After purification, bee venom samples were analysed using high-performance liquid chromatography (HPLC) equipped with a variable wavelength detector (VWD) (Agilent 1260 Series). Separation of the major venom components—apamin, phospholipase A₂, and melittin—was achieved using an InfinityLab Poroshell EC-C18 column (4.6 mm × 50 mm, 2.7 μm). The column temperature was maintained at 20 °C with a flow rate of 1 mL/min.

The mobile phase A consisted of 0.1% (v/v) formic acid in water, while the mobile phase B consisted of 0.1% (v/v) formic acid in acetonitrile. Prior to injection, samples were filtered through a 0.20 μm polytetrafluoroethylene (PTFE) membrane and transferred to vials. Absorbance was monitored at 220 nm as previously described [7].

Standard solutions of apamin (Sigma-A1289), phospholipase A₂ (Sigma-P9279), and melittin (Sigma M2272) were prepared at concentrations of 25, 50, 100, and 125 µg/mL, and four-point calibration curves were generated for component identification and quantification. All analyses were performed in triplicate.

### Cell Cultures

HCEC-12 (Human Corneal Endothelial Cells), HCE-T (Human Corneal Epithelial Cells), NTM5 (Human Trabecular Meshwork Cells), and ARPE-19 (Retinal Pigment Epithelium Cells) were cultured under standard conditions. Cells were grown in their respective media, supplemented with fetal bovine serum (FBS) and antibiotics, and maintained at 37 °C in a humidified incubator with 5% CO₂. HCEC-12 (HCEC-12 (RRID: CVCL_2064) ACC 646, DSMZ, passage 14) cells, were maintained in a 1:1 mixture of Medium 199 and Ham’s F12 (Invitrogen, UK), supplemented with 5% fetal bovine serum (FBS) (Biosera, Labtech, UK) and 1% penicillin-streptomycin (P/S) (Invitrogen, UK). HCE-T cells were donated by Kaoru Araki-Sasaki (Itami City, Japan, passage 25) [28] and were cultured in DMEM/F12 medium supplemented with 10% FBS and 1% P/S. NTM5 cells, SV40-immortalissed (NTM5) human TM cells were obtained from Alcon (Fort Worth, TX, USA, passage 22), were maintained in DMEM with low glucose, supplemented with 10% FBS and 1% P/S. ARPE-19 cells (ATCC number CRL-2302, passage 26), were cultured in DMEM/F12 medium supplemented with 10% FBS and 1% P/S. All cell culture reagents were obtained from Sigma (UK) unless otherwise stated. Cells were routinely monitored under a microscope (Nikon Eclipse) for confluency and passaged when they reached ~ 80% confluence.

### MTT Test

Cell viability was assessed using the MTT (3-(4,5-dimethylthiazol-2-yl)-2,5-diphenyltetrazolium bromide) assay. Cells (ARPE-19, NTM5, HCEC-12, and HCE-T) were seeded in 96-well plates at a density of 0.01 × 10⁶ cells per well and allowed to adhere overnight at 37 °C in a humidified incubator with 5% CO₂. Purified BV was freshly dissolved in culture medium specific to each cell line and applied at concentrations of 3.125, 6.25, 12.5, 25, 50, 100, and 200 µg/mL. Control wells received the same volume of vehicle to exclude solvent-related effects. Preliminary pilot studies indicated that higher concentrations produced near-complete cytotoxicity; therefore, the selected range was refined to capture both sublethal and cytotoxic thresholds for reliable dose–response analysis.

Treatments were carried out for 24 and 48 h. Negative controls consisted of cells cultured in medium only, while Triton X-100 (0.1%) was used as a positive control for complete cell death. Following treatment, 20 µL of MTT solution (5 mg/mL in PBS; Thermo Fisher Scientific, UK) was added to each well and incubated for 4 h at 37 °C. Metabolically active cells reduced MTT to insoluble formazan crystals, which were subsequently solubilized by adding 100 µL of dimethyl sulfoxide (DMSO) to each well. Plates were gently shaken to ensure complete dissolution, and absorbance was measured at 570 nm using a FLUOstar Optima microplate reader, with background correction at 630 nm. Cell viability was expressed as a percentage of the untreated control group. All experiments were performed in triplicate and independently repeated at least three times.

### Cytotoxicity Evaluation

Cytotoxicity was calculated relative to untreated cells (maximal viability, Max V = 100%) and Triton-X treated cells (minimal viability, Min V = 0%) using the following formula:

$${\mathrm{Cytotoxicity}}\:{(\%)} = {[{1}-{({\mathrm{Test}} - {\text{Min V}})}/{({\text{Max V}} - {\text{Min V}})}]}{\times}{100}.$$

The half-maximal inhibitory concentration (IC₅₀) was determined using a sigmoidal dose-response model. IC₅₀ values were calculated based on MTT assay data using non-linear regression analysis.

### Live/Dead Assay

Cell viability was assessed using the Live/Dead™ Viability/Cytotoxicity Kit (Thermo Fisher Scientific), following the manufacturer’s protocol. Cells were cultured in 96-well plates and, at the endpoint, washed twice with PBS. A staining solution containing 2 µM calcein-AM and 4 µM ethidium homodimer-1 (EthD-1) in PBS was freshly prepared and applied to the wells. Plates were incubated for 30 min at room temperature in the dark. After incubation, cells were gently rinsed with PBS and imaged immediately using an inverted fluorescence microscope (Zeiss Observer Z1). For each condition, at least three independent wells were analysed, and a minimum of three randomly selected fields per well were imaged using identical acquisition settings. Live cells were visualised using green fluorescence (Ex/Em: 494/517 nm), and dead cells using red fluorescence (Ex/Em: 528/617 nm).

### Image Quantification

Image analysis was performed in ImageJ (NIH). For each fluorescence channel, images were first converted to 8-bit and background subtraction was applied. The threshold was set manually (kept constant across all images) to segment live (green) and dead (red) cells. Particle analysis was conducted with size and circularity parameters set to exclude debris. The number of live and dead cells per field was quantified for each replicate.

Cell viability was calculated as:

$${\mathrm{Viability}}\:{(\%)} = {\mathrm{Number}} {\mathrm{of}} {\mathrm{live}} {\mathrm{cells}} / {({\text{Number of live}} + {\mathrm{dead}} {\mathrm{cells}})}{\times}{100}.$$

### Statistical Analysis

All experiments were performed in 3–5 independent replicates, and data are presented as mean ± standard deviation (SD). Statistical analyses were conducted using GraphPad Prism (version 8.0.2, GraphPad Software, San Diego, CA, USA). Dose response curves were fitted using non-linear regression (logarithmic dose response, variable slope) to calculate IC₅₀ values. Comparisons between multiple groups were performed using one-way analysis of variance (ANOVA) followed by Tukey’s multiple comparison test. Data distribution was assessed using the Shapiro–Wilk test, and homogeneity of variances was evaluated with Levene’s test prior to one-way ANOVA. p-value < 0.05 was considered statistically significant.

## Results

**Fig. 1:**
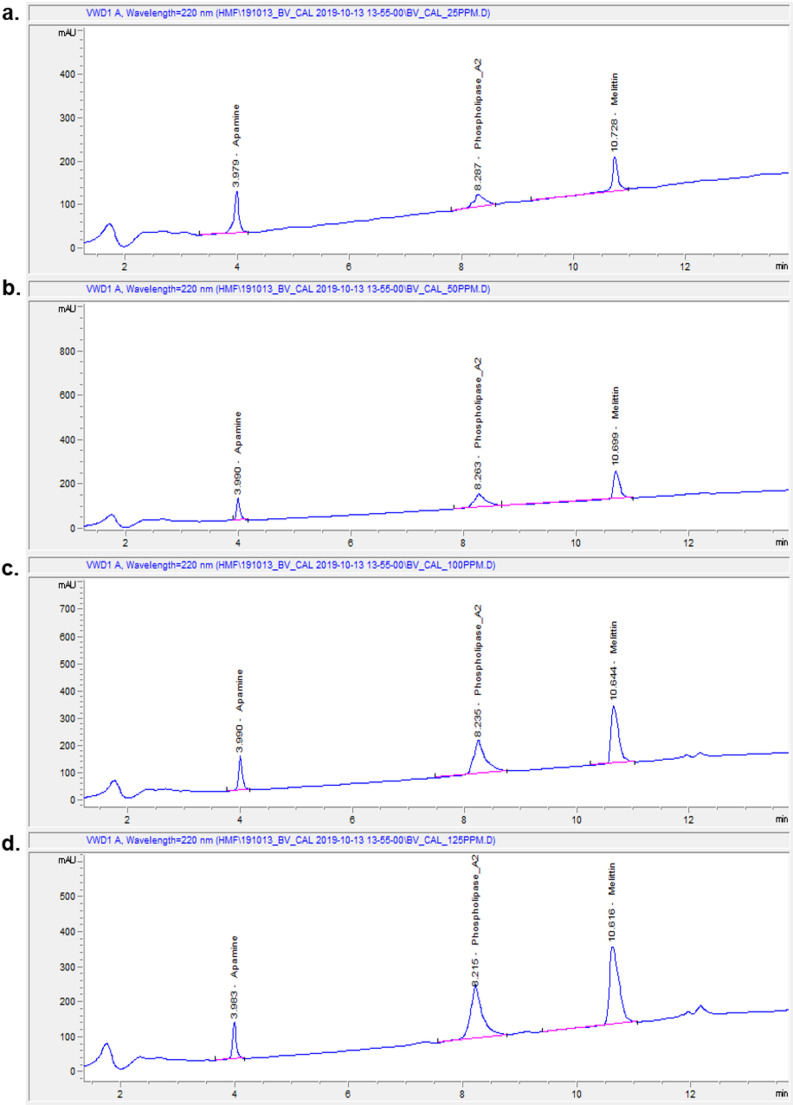
HPLC-VWD chromatograms of bee venom peptide standards. Chromatograms show separation of Apamin, Phospholipase A₂, and Melittin standards at concentrations of (a) 25 µg/mL, (b) 50 µg/mL, (c) 100 µg/mL, and (d) 125 µg/mL. Peaks were detected at 230 nm using a variable wavelength detector (VWD), and retention times were consistent with reference standards

**Fig. 2:**
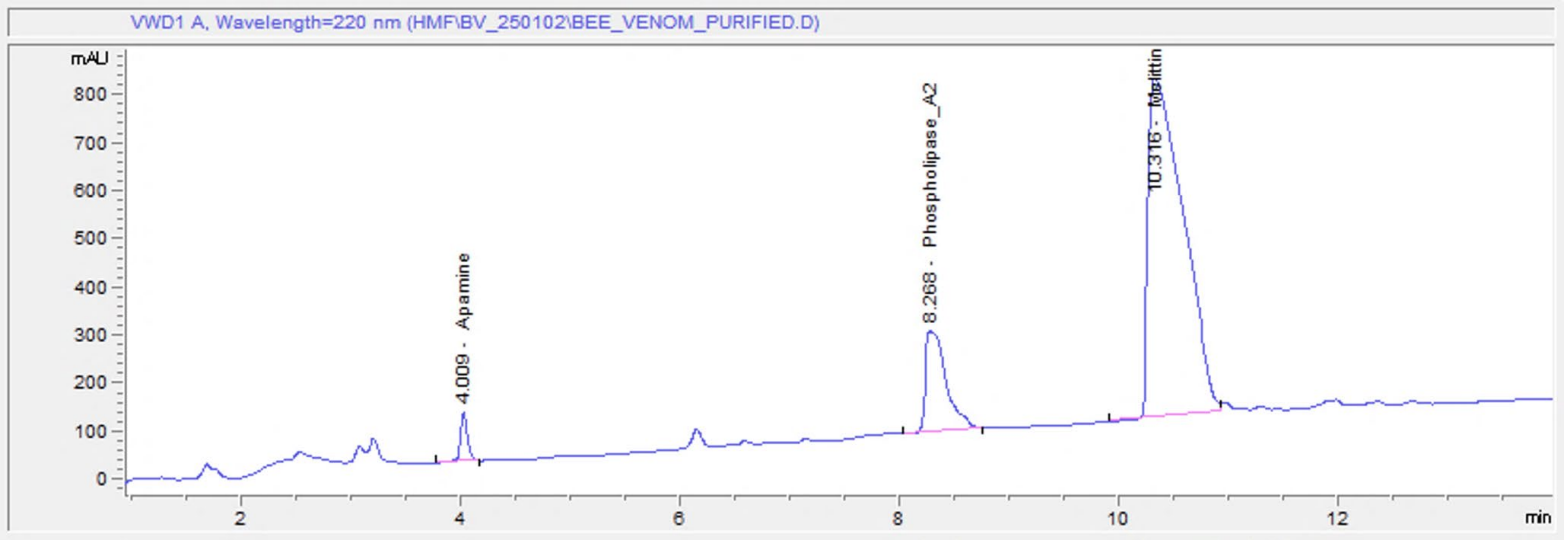
HPLC-VWD chromatogram of purified bee venom. Representative chromatogram of purified bee venom recorded at 220 nm, showing the separation of major bioactive components. Distinct peaks correspond to Apamin (retention time ~ 4.0 min), Phospholipase A₂ (~ 8.3 min), and Melittin (~ 10.1 min), identified by comparison with reference standards

Chromatographic analysis revealed three distinct peaks corresponding to apamin, phospholipase A₂, and melittin at retention times of approximately 3.9, 8.2, and 10.6 min, respectively (Fig. 1). At 25 µg/mL, the peaks for all three components were clearly separated but exhibited lower intensities. Increasing the standard concentration to 50 and 100 µg/mL resulted in proportionally higher peak areas, reflecting concentration-dependent increases in signal intensity. At 125 µg/mL, the peaks remained sharp and well-resolved, with no evidence of overlap or interference. These chromatograms confirm the reliability of HPLC-VWD for detecting and quantifying the major bioactive peptides of bee venom and provide standard reference retention times for comparison with purified venom samples. The chromatogram of purified bee venom showed three corresponding peaks at retention times of 4.0, 8.2, and 10.7 min, consistent with the reference standards (Fig. 2). In the determination of the content and composition of bee venom analysis by using HPLC-VWD, it revealed that 71.08 ± 0.33% melittin, 12.98 ± 0.27% phospholipase A_2_, and 3.02 ± 0.25% apamin in the purified bee venom. Melittin, the major component of bee venom and exceeding 71%, was one of the quality indicators. These results also demonstrated that the venom was not damaged during collection and purification. Also, this high melittin level indicated that it was free from adulteration.

**Fig. 3:**
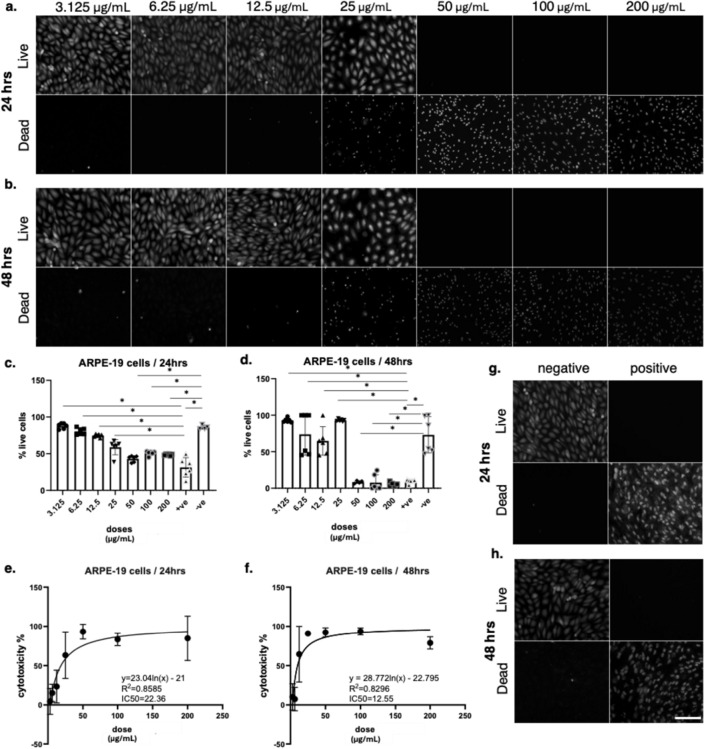
Cytotoxic effects of bee venom on ARPE-19 cells assessed by live/dead staining and viability assays. (a–b) Representative fluorescence images of ARPE-19 cells treated with increasing concentrations of bee venom (3.125–200 µg/mL) for (a) 24 h and (b) 48 h. Live cells are shown in the top panels and dead cells in the bottom panels. Scale bars = 100 μm. (c–d) Quantification of percentage live cells at 24 h (c) and 48 h (d), demonstrating a dose-dependent reduction in viability. Statistical analysis was performed using one-way ANOVA followed by Tukey’s multiple comparison test after verification of statistical assumptions, with significance set at *p < 0.001. Data are presented as mean ± SD of n = 3 independent experiments. (e–f) Non-linear regression analysis of cytotoxicity data at 24 h (e) and 48 h (f), showing IC₅₀ values of 22.36 µg/mL and 12.55 µg/mL, respectively. (g–h) Representative controls showing negative (untreated) and positive (cytotoxic) conditions at 24 h (g) and 48 h (h)

Live/dead staining revealed that BV induced a dose and time dependent cytotoxic effect on ARPE-19 cells. After 24 h, cells exposed to concentrations up to 12.5 µg/mL largely retained normal morphology and viability, whereas exposure to 25 µg/mL began to reduce the number of viable cells. At 50 µg/mL and above, substantial cell death was observed, with almost complete loss of viability at 100 and 200 µg/mL (Fig. 3). By 48 h, cytotoxicity was more pronounced, with clear reductions in live cells already visible at 12.5 µg/mL and near total cell death at concentrations ≥ 50 µg/mL. Cells in the negative control group (medium only) displayed normal morphology and high viability, while Triton X-treated cells (positive control) exhibited complete cell death at both 24 h and 48 h, validating the experimental conditions. MTT assay quantification confirmed these findings. At 24 h, ARPE-19 cell viability remained above 80% at ≤ 12.5 µg/mL but decreased significantly at 25 µg/mL and higher. At 48 h, a stronger cytotoxic effect was observed, with viability falling below 50% at 25 µg/mL and approaching baseline levels at concentrations ≥ 50 µg/mL. Statistical analysis demonstrated significant differences between treatment groups compared with controls, indicating a clear dose-dependent effect. Nonlinear regression analysis of cytotoxicity data indicated an IC₅₀ of 22.36 µg/mL at 24 h (R² = 0.8296) and 12.55 µg/mL at 48 h (R² = 0.8585).

**Fig. 4:**
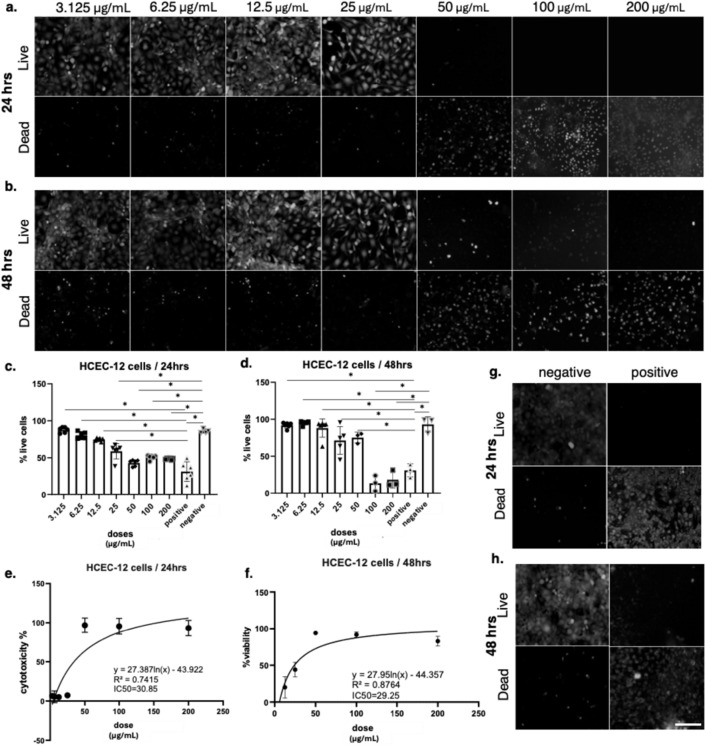
Cytotoxic effects of bee venom on HCEC-12 cells assessed by live/dead staining and viability assays. (a–b) Representative fluorescence images of HCEC-12 cells treated with increasing concentrations of bee venom (3.125–200 µg/mL) for (a) 24 h and (b) 48 h. Live cells are shown in the top panels and dead cells in the bottom panels. Scale bars = 100 μm. (c–d) Quantification of percentage live cells at 24 h (c) and 48 h (d), demonstrating a dose-dependent reduction in viability. Statistical analysis was performed using one-way ANOVA followed by Tukey’s multiple comparison test after verification of statistical assumptions, with significance set at *p < 0.001. Data are presented as mean ± SD of n = 3–5 independent experiments. (e–f) Non-linear regression analysis of cytotoxicity data at 24 h (e) and 48 h (f), showing IC₅₀ values of 30.85 µg/mL and 29.25 µg/mL, respectively. (g–h) Representative controls showing negative (untreated) and positive (cytotoxic) conditions at 24 h (g) and 48 h (h)

In addition to retinal cells, bee venom also exerted cytotoxic effects on corneal endothelial (HCEC-12) cells (Fig. 4). Live/dead staining demonstrated that at 24 h, cells exposed to concentrations up to 12.5 µg/mL largely maintained viability, whereas 25 µg/mL induced a moderate reduction in live cells. At 50 µg/mL and higher, cell death became evident, with almost complete loss of viability at 100 and 200 µg/mL. After 48 h, the toxic response was stronger, with visible reductions in viability beginning at 12.5 µg/mL, pronounced cell death at 25 µg/mL, and near-total loss of viable cells at concentrations ≥ 100 µg/mL. Negative control cells maintained normal morphology and viability, while positive control treatment with Triton X resulted in complete cell death at both 24 and 48 h, validating assay performance. MTT assay measurements confirmed these findings. At 24 h, HCEC-12viability was maintained above 80% at 12.5 µg/mL and below but decreased significantly at 25 µg/mL and higher. By 48 h, viability fell below 50% at 25 µg/mL, declining further with increasing concentrations and approaching complete loss at 100 and 200 µg/mL. Statistical analysis indicated significant differences between treatment groups and untreated controls, confirming the dose-dependent effect (q < 0.001). Dose–response curve fitting revealed IC₅₀ values of 30.85 µg/mL at 24 h (R² = 0.7415) and 29.25 µg/mL at 48 h (R² = 0.8764).

**Fig. 5:**
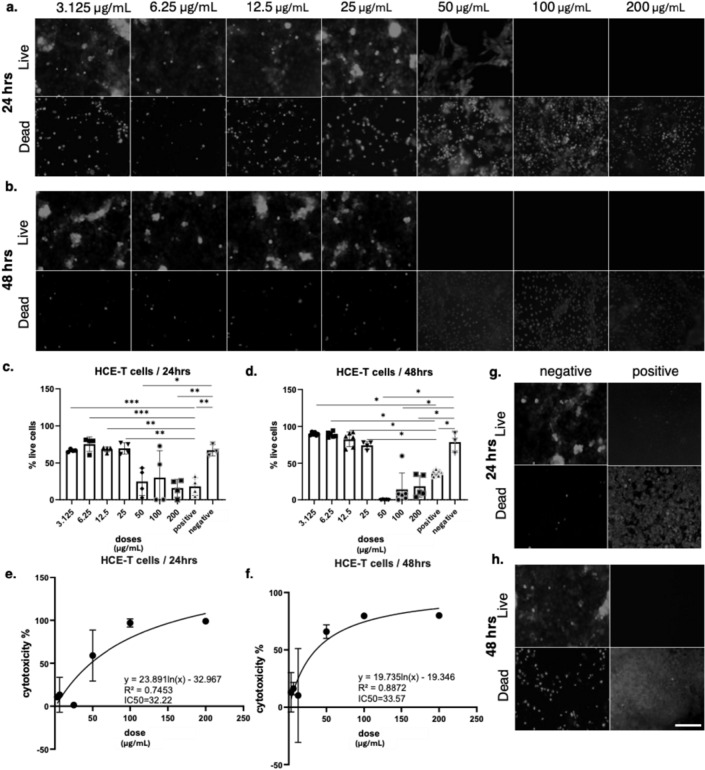
Cytotoxic effects of bee venom on HCE-T cells assessed by live/dead staining and viability assays. (a–b) Representative fluorescence images of HCE-T cells treated with increasing concentrations of bee venom (3.125–200 µg/mL) for (a) 24 h and (b) 48 h. Live cells are shown in the top panels and dead cells in the bottom panels. (c–d) Quantification of percentage live cells at 24 h (c) and 48 h (d), demonstrating a dose-dependent reduction in viability. Statistical analysis was performed using one-way ANOVA followed by Tukey’s multiple comparison test after verification of statistical assumptions, with significance indicated as *p < 0.05, **p < 0.01, and ***p < 0.001. Data are presented as mean ± SD of n = 3–5 independent experiments. (e–f) Non-linear regression analysis of cytotoxicity data at 24 h (e) and 48 h (f), showing IC₅₀ values of 32.22 µg/mL and 33.57 µg/mL, respectively. (g–h) Representative controls showing negative (untreated) and positive (cytotoxic) conditions at 24 h (g) and 48 h (h)

Similar to ARPE-19 and HCEC-12 cells, bee venom exposure produced a dose and time dependent cytotoxic effect in HCE-T corneal epithelial cells. Live/dead staining (Fig. 5) showed that at 24 h, cells remained viable up to 12.5 µg/mL, while at 25 µg/mL the number of live cells was reduced. At 50 µg/mL and higher, cytotoxicity became evident, with almost complete loss of viable cells at 100 and 200 µg/mL. After 48 h, the toxic effect was stronger, with reduced viability already observed at 12.5 µg/mL, widespread cell death at 25 µg/mL, and complete loss of viable cells at 100 and 200 µg/mL. Negative controls displayed normal morphology and viability, while positive controls treated with Triton X showed complete cell death at both 24 h and 48 h, confirming assay validity. MTT assay quantification supported these results. At 24 h, HCE-T viability stayed above 80% at concentrations ≤ 12.5 µg/mL but decreased significantly at 25 µg/mL and above. After 48 h, viability dropped below 50% at 25 µg/mL and progressively declined with higher concentrations, approaching complete cell death at 100 and 200 µg/mL. Nonlinear regression analysis showed IC₅₀ values of 32.22 µg/mL at 24 h (R² = 0.7453) and 33.57 µg/mL at 48 h (R² = 0.8872), indicating that HCE-T cells required slightly higher concentrations for cytotoxicity compared to ARPE-19 and HCEC-12 cells.

**Fig. 6:**
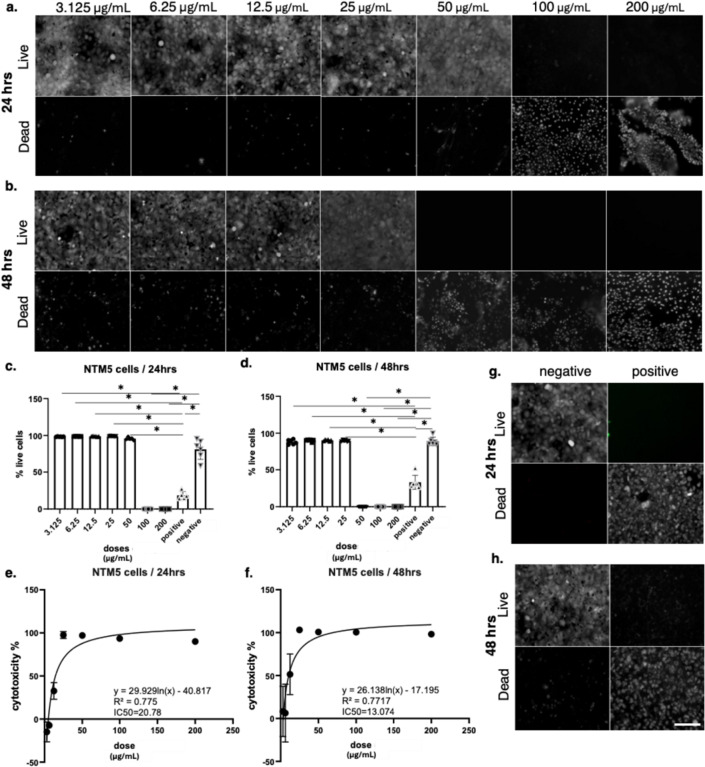
Cytotoxic effects of bee venom on NTM5 cells assessed by live/dead staining and viability assays. (a–b) Representative fluorescence images of NTM5 cells treated with increasing concentrations of bee venom (3.125–200 µg/mL) for (a) 24 h and (b) 48 h. Live cells are shown in the top panels and dead cells in the bottom panels. Scale bars = 100 μm. (c–d) Quantification of percentage live cells at 24 h (c) and 48 h (d), demonstrating a strong dose-dependent reduction in viability. Statistical analysis was performed using one-way ANOVA followed by Tukey’s multiple comparison test after verification of statistical assumptions, with significance set at *p < 0.001. Data are presented as mean ± SD of n = 3–5 independent experiments. (e–f) Non-linear regression analysis of cytotoxicity data at 24 h (e) and 48 h (f), showing IC₅₀ values of 20.78 µg/mL and 13.07 µg/mL, respectively. (g–h) Representative controls showing negative (untreated) and positive (cytotoxic) conditions at 24 h (g) and 48 h (h)

As well as ARPE-19, HCEC-12, and HCE-T cells, bee venom cytotoxicity was also examined in NTM5 cells. Live/dead staining showed that at 24 h, cells remained viable up to 12.5 µg/mL, while 25 µg/mL and higher concentrations induced noticeable reductions in live cells. At 50 µg/mL, widespread cytotoxicity was observed, and at 100 and 200 µg/mL nearly all cells were non-viable. After 48 h, the cytotoxic response was more pronounced, with cell death visible already at 12.5 µg/mL, a sharp reduction in live cells at 25 µg/mL, and almost complete cell loss at 100 and 200 µg/mL. As in previous assays, negative control cells maintained normal morphology and viability, while positive control cells treated with Triton X showed complete cell death at both 24 h and 48 h, confirming the reliability of the experimental setup. MTT assay quantification confirmed these observations. At 24 h, NTM5 cell viability remained close to 100% at concentrations ≤ 12.5 µg/mL but dropped significantly at 25 µg/mL and above (Fig. 6). After 48 h, viability decreased to below 50% at 25 µg/mL and progressively declined with increasing concentrations, reaching near-complete cell death at 100 and 200 µg/mL. Nonlinear regression analysis revealed IC₅₀ values of 20.78 µg/mL at 24 h (R² =0.775) and 13.07 µg/mL at 48 h (R² = 0.7717). These values indicated that NTM5 cells were more sensitive to bee venom than HCEC-12 and HCE-T cells, with toxicity evident at lower concentrations.

## Discussion

The present study should be considered an early ocular safety screening designed to determine whether bee venom poses cytotoxic risk to sensitive ocular tissues and to define concentration-dependent hazard thresholds rather than to evaluate therapeutic applicability. In this study, purified BV obtained from *Apis mellifera anatolica* (Muğla ecotype) was analysed for its biochemical composition and cytotoxic effects on human ocular derived cells. First chromatographic analysis of BV confirmed the presence of the three principal bioactive peptides; apamin, PLA₂, and melittin at retention times consistent with standard references. Second, the cytotoxicity assays demonstrated that BV exerted strong, dose and time dependent effects on all tested cell types, but sensitivity varied across cell lines.

Previous studies have consistently reported melittin as the predominant constituent of bee venom, typically accounting for 40–70% of the total dry weight [1]. In present analysis, the melittin content exceeded this range, representing 71.08 ± 0.33%, suggesting high venom purity and preservation of bioactive integrity during collection and processing [14]. PLA₂ was the second most abundant component at 12.98 ± 0.27%, followed by apamin at 3.02 ± 0.25%. These proportions are consistent with previous reports, which generally describe PLA₂ levels between 11 and 19% and apamin levels around 2–4% [29].

The cytotoxicity assays revealed that BV produced pronounced dose and time dependent effects in all examined cell types, though the degree of sensitivity differed between cell lines. Retinal pigment epithelial (RPE) cells form a metabolically active monolayer located between the photoreceptors and choroidal vasculature and play essential roles in visual homeostasis, immune regulation, and oxidative stress resistance. The high metabolic activity of these cells may increase their sensitivity to compounds such as bee venom [29–31]. In the present study, ARPE-19 cells showed the greatest susceptibility, with IC₅₀ values of 22.36 µg/mL at 24 h and 12.55 µg/mL at 48 h, indicating that relatively low concentrations were sufficient to impair retinal cell viability. This reduction of the IC₅₀ over time is also consistent with the live/dead staining results, which showed that at 24 h cells remained largely viable up to 25 µg/mL with clear cytotoxicity only at ≥ 50 µg/mL, whereas at 48 h visible reductions in live cells were already apparent at 12.5 µg/mL and widespread cell death occurred at concentrations ≥ 25 µg/mL. This time dependent increase in cytotoxicity reflects a common pattern in RPE cells, where prolonged exposure to otherwise sublethal concentrations can induce delayed cell damage through cumulative stress mechanisms [32]. Similar results have been reported for indocyanine green, which caused minimal effects after short exposures but induced marked cytotoxicity in RPE cells after longer exposure times [32]. Other studies also confirm that RPE toxicity is not only dose dependent but also strongly time dependent, with extended exposures lowering IC₅₀ values and amplifying cell death [33, 34]. Damage to the RPE causes various retinopathies that can also lead to vision loss. The regeneration of these cells is extremely limited, and the risk of irreversible damage after eye exposure to bee venom is a considerable issue [31]. If ocular exposure were to occur, the high concentration reaching the RPE cells may limit the safe use of therapeutic doses.

In contrast, HCEC-12 corneal endothelial and HCE-T corneal epithelial cells exhibited moderate sensitivity, with IC₅₀ values around 30–34 µg/mL. This was also reflected in the live/dead staining, where both cell types-maintained viability at concentrations up to 12.5 µg/mL and only began to show marked cytotoxicity at ≥ 25 µg/mL after 48 h. The relatively higher tolerance of these corneal-derived cells may be related to their physiological adaptation as the outermost ocular layers that are naturally exposed to environmental stressors such as UV light, oxidative damage, and pathogens, which has been shown to promote enhanced detoxification and stress-response mechanisms [35]. Corneal epithelial cells exhibit a strong capacity for self-renewal and proliferation, which may contribute to their relative resistance to bee venom toxicity [36]. However, it should also be noted that while corneal epithelial cells can regenerate, corneal endothelial cells have very limited regenerative capacity [37]. Although the IC₅₀ values indicate relatively greater tolerance compared with RPE cells under the present experimental conditions, damage to the corneal endothelium may potentially lead to clinically significant consequences given its limited regenerative capacity reported in vivo. However, such implications remain hypothetical and should be interpreted cautiously within the constraints of an in vitro model. These considerations underscore the importance of accounting for tissue-specific regenerative differences when assessing the cytotoxic potential of bee venom.

The trabecular meshwork (TM) plays a critical role in regulating aqueous humor outflow and maintaining intraocular pressure; therefore, even moderate cytotoxicity may have significant physiological consequences [38, 39]. Dysfunction of this structure is a major contributor to the pathogenesis of glaucoma, and previous studies have shown that TM cells are particularly vulnerable to oxidative stress and toxic insults [40]. In our study, NTM5 cells displayed sensitivity to bee venom, with IC₅₀ values of 20.78 µg/mL at 24 h and 13.07 µg/mL at 48 h. Live/dead staining supported these findings, showing that cells were largely viable at concentrations ≤ 12.5 µg/mL after 24 h, but marked reductions in live cells were evident at 25 µg/mL and nearly complete cell death occurred at ≥ 100 µg/mL after 48 h. The relatively low IC₅₀ values observed in this study suggest that bee venom exposure, particularly with intraocular application, could compromise TM integrity and potentially increase glaucoma risk [41]. While direct exposure of this tissue to bee venom is less likely under topical use, intraocular applications may pose a greater risk and therefore require stringent dose limitation and delivery strategies to avoid adverse outcomes.

Taken together, these results highlight distinct tissue-specific sensitivities: ARPE-19 cells were most vulnerable, followed by NTM5 cells, while HCEC-12 and HCE-T cells showed greater tolerance. From a biological perspective, this indicates that retinal and trabecular tissues are at the highest risk of bee venom toxicity, whereas the corneal epithelium and endothelium may offer a wider relative tolerance range. Nevertheless, the limited regenerative capacity of both RPE and corneal endothelium underscores the potential for irreversible damage, even at moderate concentrations. Reported IC₅₀ values for bee venom vary substantially across studies, with several investigations describing cytotoxic effects at lower concentrations [42]. Such variability is not unexpected and may reflect differences in cell type, metabolic activity, assay methodology, exposure duration, and venom composition. Notably, bee venom is a biologically heterogeneous substance whose peptide profile can vary according to geographic origin, bee species, and collection procedures. The relatively higher IC₅₀ values observed in the present study may therefore indicate differential cellular tolerance under the specific experimental conditions rather than reduced biological activity.

The concentration range evaluated in this study was intentionally selected to support screening-level hazard identification rather than to directly reflect therapeutic exposure. Given the limited data regarding ocular application of bee venom, testing a broad dose spectrum enabled characterisation of cytotoxic thresholds and dose–response relationships across multiple ocular cell types. Accordingly, these findings should not be interpreted as defining clinically relevant exposure levels.

The observed cytotoxicity is consistent with the biological properties of melittin and PLA₂, which are known to disrupt cellular membranes, increase permeability, and induce apoptosis and necrosis in a dose-dependent manner [43]. The synergistic activity of melittin and PLA₂ has been shown to amplify these cytotoxic effects [44], which likely explains the steep reductions in viability observed in ARPE-19 and NTM5 cells at relatively low doses. While these mechanisms underlie potential anticancer and anti-inflammatory applications [6], they also pose significant risks when considering direct ocular use. Indeed, clinical case reports have documented corneal edema, uveitis, optic neuropathy, and other complications following bee stings near the eye [15, 16], underscoring the relevance of our in vitro findings.

The present study also emphasises the dual nature of BV its therapeutic potential versus its toxic effects. Previous studies have highlighted its anti-inflammatory, neuroprotective, and anticancer properties [3, 5, 6], but the narrow relative tolerance range and strong cytotoxicity demonstrated here reinforce the need for precision in formulation and delivery. While bee venom has attracted scientific interest due to its bioactive properties, the pronounced cytotoxic responses observed in this study highlight the importance of cautious interpretation. Any potential biomedical application would require extensive validation in physiologically relevant models to establish an appropriate safety margin [21, 22].

This work provides important insights into the safety profile of Anatolian bee venom on ocular cells, but several limitations must be noted. The assays were performed in vitro, and cellular responses may differ in vivo due to systemic factors, immune interactions, and tissue repair mechanisms. The experiments were conducted exclusively in two-dimensional immortalised ocular cell lines, which, although widely used for preliminary toxicological screening, do not fully replicate the structural and functional complexity of the human eye. Advanced models such as three-dimensional reconstructed human corneal epithelium (e.g., OECD Test Guideline 492) or primary ocular cells may provide greater physiological relevance for ocular hazard identification and risk assessment. Therefore, the present findings should be interpreted as exploratory and hypothesis-generating, warranting further investigation in higher-tier experimental systems. Additionally, we examined crude purified venom as a whole, whereas individual components (e.g., melittin, PLA₂) or modified peptides may exert different toxicity and therapeutic profiles. Future work should focus on fractionating BV components, testing targeted delivery systems, and evaluating dose–response effects in animal models of ocular disease.

An additional limitation should be considered. Although the venom was handled under controlled conditions, endotoxin levels were not specifically quantified. Trace endotoxin contamination could theoretically influence cellular responses, particularly in sensitive in vitro systems. Therefore, future studies incorporating endotoxin testing (e.g., LAL assay) would further strengthen the toxicological interpretation of these findings.

In summary, purified *Apis mellifera anatoliaca* venom contained high-quality melittin and exhibited potent, dose-dependent cytotoxic effects on ocular cell lines, with retinal pigment epithelium and trabecular meshwork cells exhibiting heightened vulnerability. These results reinforce the need for cautious evaluation of BV in ocular applications, highlight the importance of tissue-specific responses, and suggest that controlled delivery approaches are essential to balance safety with therapeutic potential.

## Data Availability

No datasets were generated or analysed during the current study.

## Declarations

This study did not involve human participants, human data, or live vertebrate animals.
